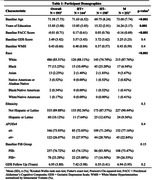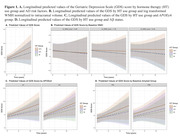# Examining the impact of hormone therapy and Alzheimer’s disease risk factors on depressive symptoms in the Harvard Aging Brain Study

**DOI:** 10.1002/alz.094828

**Published:** 2025-01-09

**Authors:** Kelly A. Lopez Garcia, Hannah M Klinger, Michael J Properzi, Jennifer R. Gatchel, Gad A Marshall, Nancy J Donovan, Zahra Shirzadi, Aaron P Schultz, Jasmeer P. Chhatwal, Reisa A Sperling, Gillian T Coughlan, Rachel F Buckley

**Affiliations:** ^1^ Massachusetts General Hospital, Harvard Medical School, Boston, MA USA; ^2^ Massachusetts General Hospital, Boston, MA USA; ^3^ Department of Neurology, Massachusetts General Hospital, Harvard Medical School, Boston, MA USA; ^4^ Department of Psychiatry, Massachusetts General Hospital, Harvard Medical School, Boston, MA USA; ^5^ Brigham and Women’s Hospital, Harvard Medical School, Boston, MA USA; ^6^ Brigham and Women’s Hospital, Boston, MA USA; ^7^ Massachusetts General Hospital, Harvard Medical School, Department of Neurology, Boston, MA USA; ^8^ Brigham and Women’s Hospital and Department of Neurology, Massachusetts General Hospital, Harvard Medical School, Boston, MA USA

## Abstract

**Background:**

Hormone therapy (HT) is often used to manage symptoms related to menopause, but its longer‐term effects on depressive symptoms in older women remains unclear. Previous literature reports inconclusive results on whether HT use is protective against or associated with increased depressive symptoms over time in older women. The objective of this study was to examine the associations of self‐reported HT use with baseline and longitudinal later life depressive symptoms. We also investigated whether Alzheimer’s disease (AD) risk factors such as *APOE*e4 status, white matter hyperintensities (WMH), and Aβ status moderated the association between HT use and depressive symptoms.

**Method:**

We identified 593 participants from the Harvard Aging Brain Study and ancillary studies (58% Female, Age_mean(SD)_ = 72(8) years) with longitudinal data for the 30‐item Geriatric Depression Scale (GDS) and baseline self‐reported HT use for women. A subset of 355 participants had baseline Aβ status available and 266 participants had baseline WMH MRI data available. Linear regressions and linear mixed‐effects models examined the associations between baseline HT use and baseline and longitudinal GDS, using HT users as our reference group. We also tested the interaction of baseline HT use with 1. *APOE*e4 status, 2. Log transformed baseline WMH normalized to intracranial volume, and 3. Baseline Aβ status on longitudinal GDS. All models adjusted for age, education, and ethnicity.

**Result:**

42% of women reported past or current HT use. HT use was not associated with GDS at baseline. HT users reported significantly greater depressive symptoms over time relative to men (β = 0.11, p = 0.035) and exhibited a sub‐threshold trend of higher depressive symptoms relative to non‐users (β = 0.10, p = 0.069). *APOE*e4, WMH, and Aβ status did not modify the effect of HT status on longitudinal GDS.

**Conclusion:**

While our study revealed that HT users exhibit increased depressive symptoms over time relative to men, further research is needed to confirm this trend relative to non‐users and to better understand how AD risk factors moderate this relationship. Our findings are limited by lack of information on HT use, such as type, dose, and duration, which should be considered to provide insight into the complexities of women’s brain health in aging populations.